# Traumatic sternal fractures

**DOI:** 10.1590/0100-3984.2022.55.4e3

**Published:** 2022

**Authors:** Bruno Hochhegger, Stephan Altmayer

**Affiliations:** 1 University of Florida, Gainesville, FL, USA. Email: brunohochhegger@gmail.com.; 2 Stanford University, Stanford, CA, USA.

Traumatic sternal fractures are found in up to 8% of patients with blunt chest trauma and 18% of polytrauma patients with thoracic injuries, whereas cases secondary to penetrating trauma are more rare^(1,2)^. Sternal fractures most commonly result from blunt, anterior chest-wall trauma and deceleration injuries, with a reported incidence of 3.0-6.8% in motor vehicle collisions. Sports injuries, falls, and assaults account for most of the remaining cases^(1-4)^. Such fractures typically result from a direct blow to the anterior chest wall or from forced deceleration. As previously reported^(3)^, the leading mechanism is motor vehicle collision (in 68% of patients), followed by falls (in 7.9%), motorcycle accidents (in 7.9%), pedestrian versus motor vehicle accidents (in 3.4%), and cycling accidents (in 1.4%). The introduction of seat belt legislation requiring shoulder restraints has led to an increased incidence of sternal fractures^(5,6)^. In a review of 1,867 patient records in the National Israeli Trauma Registry, Odell et al.^(7)^ found that none of the patients with isolated sternal fracture required endotracheal intubation, chest tube insertion, or thoracotomy, compared with 16.9% of the polytrauma patients with sternal fractures. Isolated sternal fractures are rarely associated with blunt cardiac injury^(8)^ and have a low (0.8%) mortality rate^(9)^. Conversely, polytrauma patients with sternal fractures often have severe associated injuries, with reported mortality rates of up to 7.9%^(3)^. Chest X-ray is usually the initial imaging examination in patients with a suspected sternal injury. An anteroposterior X-ray has been shown to be only 50% sensitive for detecting sternal fractures. An X-ray obtained in a lateral view has greater sensitivity and is typically diagnostic, because most sternal fractures are transverse and any displacement occurs in the sagittal plane^(2)^. Computed tomography (CT) continues to be the gold standard for the diagnosis of sternal fracture and has been shown to be superior to lateral X-ray^(9)^. In a large retrospective study of patients with thoracic trauma, 94% of sternal fractures were visible only on chest CT^(6)^. In addition, a CT scan detects associated thoracic injuries in over 80% of patients with traumatic sternal fracture^(6)^. The degree of sternal fracture displacement is not necessarily associated with blunt cardiac injury^(10)^. Given the increase in the number of traumatic sternal fractures diagnosed, it is important for radiologists to understand the clinical significance of these injuries.

In the previous issue of Radiologia Brasileira, Şimşek et al.^(11)^ described a cohort of 108 patients with sternal fractures. The fracture was located exclusively in the manubrium in 64 patients (59.3%), exclusively in the body of the sternum in 41 (38.0%), and in both locations in three (2.7%). Morbidity rates were higher in the patients with fractures of the manubrium than in those with fractures of the body of the sternum, as was the incidence of accompanying bone fractures and organ injuries. The authors stated that fracture of the manubrium can be indicative of the severity of trauma and of a poor prognosis. In addition, mortality was significantly higher among the patients with comminuted fractures than among those with other types of fractures. These data expand the current understanding of sternal fractures and are important for this fundamental imaging diagnosis, especially in polytrauma patients.

## References

[1] Knobloch K, Wagner S, Haasper C (2006). Sternal fractures occur most often in old cars to seat-belted drivers without any airbag often with concomitant spinal injuries: clinical findings and technical collision variables among 42,055 crash victims. Ann Thorac Surg.

[2] Brookes JG, Dunn RJ, Rogers IR (1993). Sternal fractures: a retrospective analysis of 272 cases. J Trauma.

[3] Oyetunji TA, Jackson HT, Obirieze AC (2013). Associated injuries in traumatic sternal fractures: a review of the National Trauma Data Bank. Am Surg.

[4] Khoriati AA, Rajakulasingam R, Shah R (2013). Sternal fractures and their management. J Emerg Trauma Shock.

[5] Budd JS (1985). Effect of seat belt legislation on the incidence of sternal fractures seen in the accident department. Br Med J (Clin Res Ed).

[6] Perez MR, Rodriguez RM, Baumann BM (2015). Sternal fracture in the age of pan-scan. Injury.

[7] Odell DD, Peleg K, Givon A (2013). Sternal fracture: isolated lesion versus polytrauma from associated extrasternal injuries-analysis of 1,867 cases. J Trauma Acute Care Surg.

[8] Dua A, McMaster J, Desai PJ (2014). The association between blunt cardiac injury and isolated sternal fracture. Cardiol Res Pract.

[9] Kim EY, Yang HJ, Sung YM (2012). Sternal fracture in the emergency department: diagnostic value of multidetector CT with sagittal and coronal reconstruction images. Eur J Radiol.

[10] Heidelberg L, Uhlich R, Bosarge P (2019). The depth of sternal fracture displacement is not associated with blunt cardiac injury. J Surg Res.

[11] Şimşek S, Özmen CA, Onat S (2022). Morbidity and mortality associated with fracture of the sternum due to blunt trauma, by fracture type and location. Radiol Bras.

